# Exploration of associations between deliberate self-poisoning and psychiatric disorders in rural Sri Lanka: A case-control study

**DOI:** 10.1371/journal.pone.0255805

**Published:** 2021-08-06

**Authors:** P. H. G. J. Pushpakumara, A. H. Dawson, A. M. P. Adikari, S. U. B. Thennakoon, Ranil Abeysinghe, T. N. Rajapakse

**Affiliations:** 1 Department of Family Medicine, Faculty of Medicine and Allied Sciences, Rajarata University of Sri Lanka, Saliyapura, Sri Lanka; 2 SACTRC, Faculty of Medicine, University of Peradeniya, Peradeniya, Sri Lanka; 3 Central Clinical School, University of Sydney, Sydney, Australia; 4 Psychiatry Unit, Teaching Hospital Kurunegala, Kurunegala, Sri Lanka; 5 Department of Community Medicine, Faculty of Medicine, University of Peradeniya, Peradeniya, Sri Lanka; 6 Department of Psychiatry, Faculty of Medicine, University of Peradeniya, Peradeniya, Sri Lanka; St. Paul’s Hospital Millenium Medical College, ETHIOPIA

## Abstract

Psychiatric disorders are important predictors of deliberate self-harm. The present study was carried out to determine the associations between DSM-IV TR Axis- I & II disorders and deliberate self-poisoning (DSP) in a rural agricultural district in Sri Lanka. Patients residing in the district who presented with DSP were randomly selected for the study. Both the cases and age, sex, and, residential area, matched controls were assessed for DSM-IV TR Axis- I & II disorders based on the Structured Clinical Interview for DSM-IV-TR Axis I and II Disorders (SCID I & II) conducted by a specialist psychiatrist. Cases consisted of 208 (47.4%) males and 231 (52.6%) females. More than one third (37%) of males and more than half (53.7%) of females were aged below 20 years. DSM-IV TR axis-I and/or II psychiatric diagnoses were diagnosed in 89 (20.3%) of cases and 14 (3.2%) controls. Cases with a DSM-IV TR axis-I diagnosis were older than the cases without psychiatric diagnosis (32 and 19 years), p<0.0001. Having a depressive episode was associated with a 19 times higher risk for DSP. Being a male aged > = 30 years and having an alcohol use disorder carried a 21 times excess risk for DSP. A fivefold excess risk for DSP was found among 10–19 year old females with borderline personality traits. Depressive disorder and alcohol-related disorders were significantly associated with the older participants who presented with DSP. The overall prevalence of psychiatric disorders associated with DSP in rural Sri Lanka was significantly lower compared to the rates reported in the West and other countries in the region. Therefore, health and research priorities to reduce self-harm in Sri Lanka should focus both on psychiatric and non-psychiatric factors associated with DSP.

## Introduction

Deliberate self-harm (DSH) is a global health issue that is responsible for about 800,000 deaths each year [1]. The rates of DSH is 10 to 40 times greater than suicide [2, 3]. Deliberate self-poisoning (DSP) is the predominant (> 80%) reason for DSH admission to the hospitals in Sri Lanka [4]. And rural areas in Sri Lanka have had a persistently high incidence of deliberate self-poisoning (DSP) since the early 1980s [5–7]. The high rates of self-harm in Sri Lanka are multifactorial, with both cultural and environmental factors playing a role [8].

A major psychiatric diagnosis is a significant predictor of DSH [9]. The prevalence of 89.6% seen in Western countries is much higher than 70.6% reported in non-Western countries [10]. In contrast to the very high rate of DSH, a low prevalence of psychiatric co-morbidity, < 20%, has been reported in Sri Lanka [11]. And, it was much lower than the prevalence reported by countries in the Indian subcontinent [12–15]. A substantial heterogeneity between study estimates have been observed in all the regions including Sri Lanka [10, 11, 16].

Methodological deficiencies of non-Western studies have been reported. Most previous research in Asia has focused on the association of single psychiatric diagnosis with DSH [10]. Out of the studies conducted in low and middle-income countries, less than 5% of studies were prospective and employed a standardized interview schedule to obtain a diagnosis [17]. The association of personality disorders with DSH is a relatively less explored area in Asia. Similarly, a systematic review of studies on self-harm in Sri Lanka identified no studies that investigated the relative contribution or possible associations between DSH and psychiatric conditions such as anxiety disorders, impulse control disorders, or bipolar disorder [11]. Most previous Sri Lankan studies of psychiatric disorders in DSH have been based upon self-report, use of screening tools, or review of medical records [11].

Health care planning for DSH in Sri Lanka needs to consider the relative contribution of psychiatric disorders and non-psychiatric factors but this has not been previously studied. The present study was designed to address a deficit in Sri Lankan and Asian DSH research by determining the overall contribution of psychiatric disorders and DSM-IV TR Axis- I & II disorders for DSP.

## Methods

### Study design and study setting

A case-control study matching age, sex, and residential divisional secretariat division was conducted among randomly selected patients who were admitted to Teaching Hospital Kurunegala over eighteen months, from 1^st^ July 2011 to 31^st^ December 2013, as a substudy of the study titled “A clustered randomise control trial of educational interventions on treatment of patients with acute poisoning in rural Asian hospitals” (Sri Lanka Clinical Trial Registry No. SLCTR/2010/008). Teaching Hospital Kurunegala is the main referral center for the 1.6 million population of the district [18], and, receives more than 53% of the total deliberate self-poisoning cases of the district [7].

### Procedure

#### Sample and sampling

Patients admitted due to DSP, aged 10 years and above, who reside in the Kurunegala District, admitted to Teaching Hospital Kurunegala as direct admissions and as referrals, were included for the study as cases. Patients who were recruited to the study on a previous admission and deaths during the admissions were excluded. The sample size was calculated for hypothesis testing for an odds ratio, with a level of 5% significance and a two-sided test with a power of 90%. The sample size was calculated to detect a condition that has a 6% prevalence among the non-DSP population and minimum odds of two or a condition that has a 1% prevalence among the non-DSP population and minimum odds of 3.75 [19]. Four hundred and thirty-nine DSP patients, were randomly selected as cases from blocks of seven consecutively admitted consenting DSP patients, aged 10 years and above, using a computer program. The same number of individuals matching age, sex, and residential divisional secretariat division, who have no history of self-harm, were randomly selected as controls from patients presenting to the Out Patient Department, Teaching Hospital Kurunegala. As medical comorbidity is rare in rural Sri Lankan DSP patients, controls were selected from Outpatient Department rather than inpatients.

#### Study instruments and data collection

A specialist psychiatrist interviewed all participants (cases and controls) using the SCID I & II to diagnose any major psychiatric disorders and/or personality disorders (DSM-IV-TR Axis I and II disorders). Collateral history was obtained from family members. The diagnoses were recorded according to DSM-IV [20]. Interviews were conducted when the participants were medically stable, usually the day before discharge in cases, and after the Outpatient Department consultation in controls. Details related to the previous consultations of a health professional for the symptoms related to the diagnosis were collected through an interview with the patient and family members, and confirmed by examination of previous medical records.

### Data analysis

Data were entered into a Microsoft Excel spreadsheet and analyzed using IBM SPSS Statistics for Windows, Version 23.0. Medians, quartiles, and percentages with a confidence interval at a 95% significance level were calculated to describe variables. Odds ratios and confidence intervals at 95% significance level were calculated for categorical data. Binary logistic regression analysis was done to develop a predictor model for DSP behavior. Model was developed including age arranged as a binary variable (<30 and > = 30 years), sex and DSM-IV-TR Axis I and II disorders.

### Ethics statement

Ethics approval for “A clustered RCT of educational interventions on treatment of patients with acute poisoning in rural Asian hospitals” was obtained from Ethical Review Committee, Faculty of Medicine, University of Peradeniya (EC/2007/98, https://med.pdn.ac.lk/admin/committees_Ethical.html). Ethical approval for the sub-study was obtained from the Ethics Review Committee, Faculty of Medicine and Allied Sciences, Rajarata University of Sri Lanka (8/3/2011, http://www.rjt.ac.lk/med/faculty-structure/committees/erc). Informed written consent was obtained from the participants for all the interviews and to obtain the collateral history from a third party. The ascent from the participant and consent from a parent or guardian were taken for minors, aged below 18 years.

## Results

**Fig 1:**
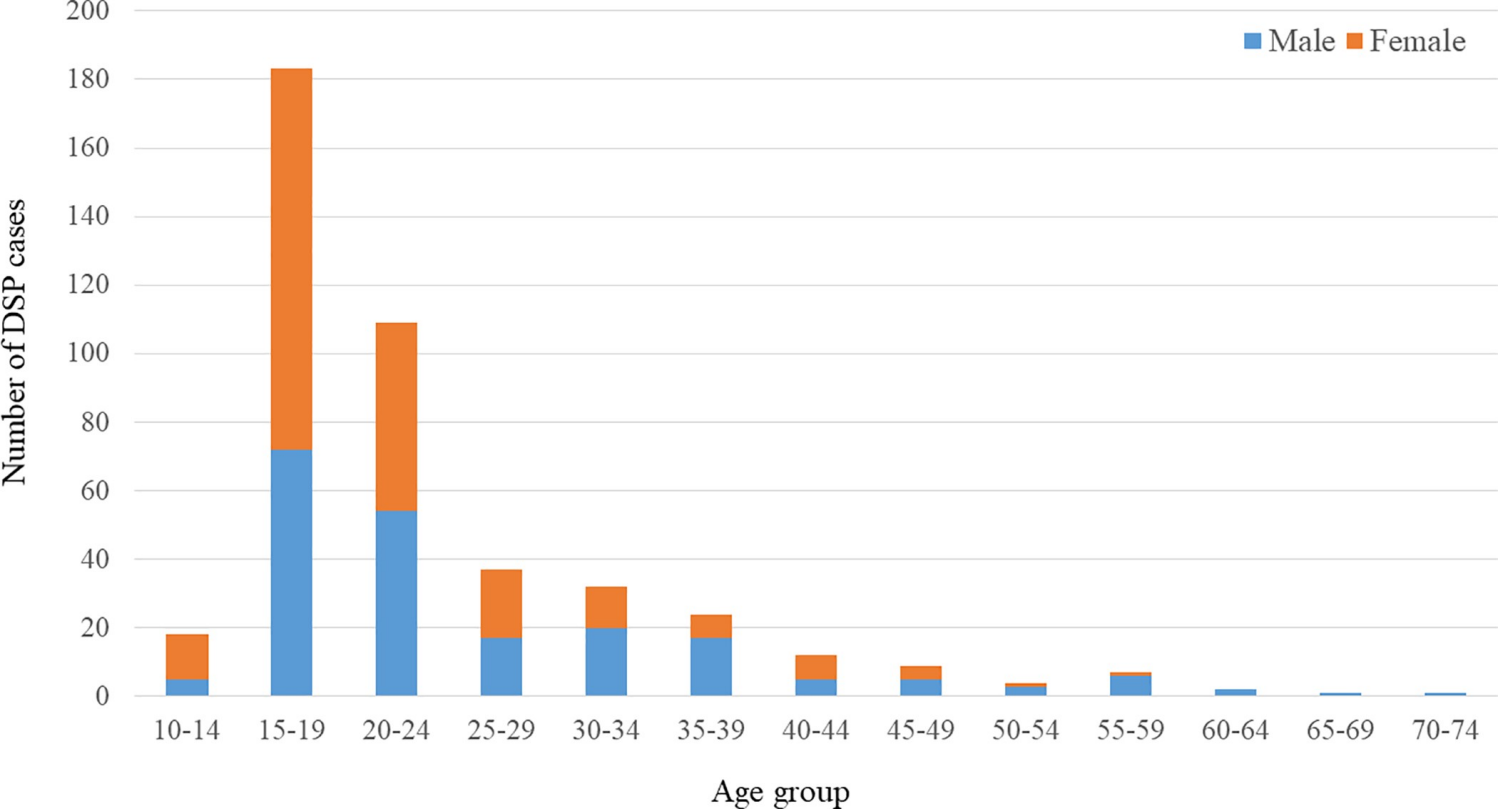
Number of cases by sex and age. The sample consisted of 47.4% males and 52.6% females, and 37% of males and 53.7% of females were aged below 20 years. About one-third of the sample was in the 20–29 year age group (male: 34.1% and female: 32.5%).

A total of 1954 and 2049 persons were admitted for DSP in 2011 and 2012 respectively. The sample consisted of 208 (47.4%) males and 231 (52.6%) females, ranging from 12 to 70 years of age (median 20, IQR 17–27) (Table 1). More than one third (37%) of males and more than half (53.7%) of females were aged below 20 years (Fig 1). Males were older than females, the median age for the males and females was 22 years (IQR 18–32) and 19 years (IQR 17–24) respectively, p<0.0001 (Mann-Whitney U test).

**Table 1:** Sociodemographic characteristics of the sample.

		Case		Control	
		Male	Female	Male	Female
n		208	231	208	231
Age: Median (IQR)		22 (18–32)	19 (17–24)	22 (18–30.75)	19 (17–23)
Ethnic Group					
n (%)	Sinhalese	201 (96.6)	222 (96.1)	203 (97.6)	216 (93.5)
	Sri Lanka Tamil	5 (2.4)	6 (2.6)	0 (0.0)	2 (0.9)
	Indian Tamil	0 (0.0)	1 (0.4)	0 (0.0)	0 (0.0)
	Moor	2 (1.0)	2 (0.9)	5 (2.4)	13 (5.6)
Religion					
n (%)	Buddhist	198 (95.2)	221 (95.7)	202 (97.1)	215 (93.1)
	Hindu	5 (2.4)	7 (3.0)	0 (0.0)	2 (0.9)
	Islam	2 (0.9)	2 (0.9)	5 (2.4)	13 (5.6)
	Roman catholic	3 (1.4)	1 (0.4)	1 (0.5)	1 (0.4)
Marrital status					
n (%)	Never Married	123 (59.1)	147 (63.6)	135 (64.9)	147 (63.6)
	Married (Registered)	75 (36.1)	65 (28.1)	70 (33.6)	65 (28.1)
	Married (Customary)	4 (1.9)	10 (4.3)	1 (0.5)	10 (4.3)
	Seperated	5 (2.4)	7 (3.0)	0 (0.0)	7 (3.0)
	Divorced	1 (0.5)	0 (0.0)	0 (0.0)	0 (0.0)
	Widowed	0 (0.0)	2 (0.9)	2 (0.9)	2 (0.9)
Level of education					
n (%)	No formal education	2 (0.9)	2 (0.9)	0 (0.0)	1 (0.4)
	< Gade 5	12 (5.8)	3 (1.3)	2 (0.9)	3 (1.2)
	Grade 5–11	149 (71.6)	139 (60.1)	92 (44.2)	95 (41.1)
	G.C.E. (O/L)	30 (14.4)	57 (24.7)	59 (28.4)	68 (29.4)
	G.C.E. (A/L)	13 (6.2)	29 (12.6)	54 (25.9)	61 (26.4)
	Diploma /Degree	2 (0.9)	1 (0.4)	1 (0.5)	3 (1.3)
	Postgraduate	0 (0.0)	0 (0.0)	0 (0.0)	0 (0.0)

A DSM-IV TR axis-I and/or II psychiatric diagnosis was present in 89 (20.3%, 95% CI 16.5–24.0%) of cases and 14 (3.2%, 95% CI 1.5–4.8%) controls. More than one psychiatric condition was found in 14 (3.2%, CI 1.5–4.8%) of cases and none of the controls. Having a psychiatric disorder was associated with a 7.7 times higher risk for DSP (OR 7.7, 95% CI 4.3–13.8, p <0.0001 Chi-square test). Cases who had at least one DSM-IV TR axis-I diagnosis (median age 32 years, IQR 20.75–43) were older than those who had no psychiatric disorder (median age 19 years, IQR 17–24), p<0.0001 (Mann-Whitney U test). The prevalence of depressive episodes among the patients who presented with DSP was 8.4% (95% CI 3.5–13.6%). Suffering from a depressive episode was shown to have 13 times excess risk for DSP (OR 13.4, 95% CI 4.1–43.7, p <0.0001 Chi-square test). The majority, 76.4% (95% CI 68.9–83.9) had never previously sought medical care for the symptoms related to their psychiatric diagnosis (Table 2).

**Table 2:** Presence of DSM-IV TR axis-I and II psychiatric disorders among cases.

Diagnosis	n (%, 95% CI)	Newly diagnosed:
		n (%, 95% CI)
Alcohol Dependence	9 (2.0, 0.7–3.3)	7 (77.8, 63.9–91.6)
Alcohol abuse	13 (3.0, 1.4–4.6)	11 (84.6, 78.2–90.9)
Poly-substance dependence	1 (0.2, -0.2–0.6)	1 (100)
Mild Depressive disorder, single episode	5 (1.1, 0.1–2.0)	5 (100)
Moderate Depressive disorder, single episode	12 (2.7, 1.2–4.2)	9 (75, 67.3–82.6)
Severe Depressive disorder without psychotic features, single episode	13 (3.0, 1.4–4.6)	11 (84.6, 78.2–90.9)
Recurrent Moderate Depressive disorder	3 (0.7, -0.1–1.5)	0 (0)
Recurrent Severe Depressive disorder without psychotic features	2 (0.5, -0.2–1.2)	1 (50, 14.6–85.4)
Recurrent Severe Depressive disorder with psychotic features	1 (0.2, -0.2–0.6)	1 (100)
Bipolar Affective Disorder-most recent episode moderate depression	1 (0.2, -0.2–0.6)	1 (100)
Schizophrenia	2 (0.4, 2.1–5.8)	0 (0)
Acute Stress Disorder	1 (0.2, -0.2–0.6)	1 (100)
Specific phobia	2 (0.5, -0.2–1.2)	1 (50, 14.6–85.4)
Moderate Mental Retardation	1 (0.2, -0.2–0.6)	0 (0)
Borderline Personality Disorder or traits	32 (7.3, 4.5–9.7)	25 (78.1, 70.8–85.4)
Antisocial Personality Disorder or traits	3 (0.7, -0.1–1.5)	2 (66.7, 39.5–93.9)
Narcissistic Personality Disorder or traits	1 (0.2, -0.2–0.6)	1 (100)
Depressive Personality Disorder or traits	2 (0.5, -0.2–1.2)	0 (0)
Obsessive Compulsive Personality Disorder or traits	0 (0)	0
Any DSM-IV TR axis I or II Disorder	89 (20.3, 16.5–24.0)	68 (76.4, 68.9–83.9)

### Alcohol use disorders

Alcohol use disorders were seen predominately amongst males aged > = 30 years (26.7%). In this age group, alcohol use disorders had an odds ratio of 21.4 for DSP. (Table 2) Cases who had alcohol use disorders (median age 35.5 years, IQR 28.5–42) were older than cases who had no alcohol use disorders (median age 20 years, IQR 17–25), p<0.0001 (Mann-Whitney U test). Only 20% of patients (n = 4) with alcohol-related problems had been diagnosed before the event of DSP. (Table 3)

**Table 3:** Presence of alcohol use disorders among DSP cases and controls by age group.

Age Group (years)	Male			Female	
	n, % (95% CI) having alcohol use disorder		OR*	n, % (95% CI) having alcohol use disorder	
			(95% CI)		
	Case	Control	p value**	Case	Control
10–19	2, 2.6	1, 1.3	2.0 (0.18–22.84),	1, 0.8	1, 0.8
	(0.8–4.4)	(0.01–2.6)	1.0	(0.003–1.6)	(0.003–1.6)
20–29	3, 4.2	1, 1.4	3.1 (0.31–30.44),	0	1, 1.3
	(1.8–6.6)	(0.01–2.8)	0.62		(0.01–2.7)
> = 30	16, 26.7	1, 1.7	21.4 (2.74–168.0),	0	0
	(20.9–32.4)	(0.01–3.3)	< 0.0001		

* unadjusted odds ratios

** p value of Fisher’s exact test.

### Depressive disorders

Depressive episodes were the commonest in the > = 30 year age group, with the gender distribution being 20% among males and 28% among females. Cases with depression were older (median age 35 years, IQR 20.5–45) than the cases without depression (median age 20 years, IQR 17–25), p<0.0001 (Mann-Whitney U test). In cases aged > = 30 years with depression, the odds ratio for DSP was significantly higher in females than in males. (Table 4) Only 24.3% of those with depression had been previously diagnosed. (Table 2)

**Table 4:** Presence of depressive episodes among DSP cases and controls by age group.

Age Group (years)	Male			Female		
	n, % (95% CI) having depressive episode		OR*	n, % (95% CI) having depressive episode		OR*
			(95% CI)			(95% CI)
	Case	Control	p value**	Case	Control	p value**
10–19	2, 2.6 (0.8–4.4)	0	5.1	4, 3.2 (1.6–4.8)	0	9.3
			(0.2 to 108.8)			(0.5 to 174.7)
			0.49			0.12
20–29	5,7.0 (4.0–10.1)	0	11.8	5, 6.7 (3.8–9.5)	0	11.8
			(0.6 to 218.2)			(0.6 to 217.1)
			0.058			0.058
> = 30	12, 20.0 (14.8–25.2)	3, 5.0 (2.2–7.8)	4.7	9, 28.1 (20.2–36.1)	0	26.3
			(1.3 to 17.8)			(1.5 to 474.5)
			0.02			0.002

* unadjusted odds ratios

** p value of Fisher’s exact test.

### Borderline personality disorder and borderline personality traits

The prevalence of borderline personality disorder or borderline personality traits among the persons who presented with DSP did not significantly differ in age groups. Age was not a predictor for diagnosis: the median age of those with borderline personality disorder or borderline personality traits was 20.5 years (IQR 16.25 to 30.00), and the median age for the others was 20.0 years (IQR 17.0 to 26.0), p = 0.8 (Mann-Whitney U test). A five-fold excess risk for DSP was found among the 10–19 years old females with borderline personality disorder or borderline personality traits (OR 5.3, 95% CI 1.1–24.9, p = 0.03, Fisher’s exact test). (Table 5) Only 21.8% patients had sought medical care for symptoms related to personality disorder before the event. (Table 2)

**Table 5:** Presence of borderline personality disorder (BPD) or borderline personality traits (BPT)among DSP cases and controls by age group.

Age Group (years)	Male			Female		
	n, % (95% CI) having BPD or BPT		OR* (95% CI) p value**	n, % (95% CI) having BPD or BPT		OR* (95% CI) p value**
	Case	Control		Case	Control	
10–19^#^	5, 6.5 (3.7–9.3)	1, 1.3 (0.01–2.6)	5.3	10, 8.1 (5.6–10.5)	0	5.3
			(0.6 to 46.3)			(1.1 to 24.9)
			0.21			0.03
20–29^##^	3, 4.2 (1.8–6.6)	0	7.3	5, 6.7 (3.8–9.5)	0	11.8
			(0.4 to 144.2)			(0.6 to 217.1)
			0.24			0.058
> = 30^##^	4, 6.7 (3.4–9.9)	0	9.6	5, 15.6 (9.2–22.0)	1, 3.1 (0.05–6.2)	5.7
			(0.5 to 183.2)			(0.6 to 52.3)
			0.12			0.19

* unadjusted odds ratios

** p value of Fisher’s exact test

^#^ BPT

^##^ BPD.

### Backward stepwise (conditional) binary logistic regression analysis

Backward stepwise (conditional) binary logistic regression analysis showed that; the presence of depressive disorder, alcohol use disorder, borderline personality disorder or borderline personality traits, and age below 30 years, were independent predictors of DSP behavior. The logistic regression model was statistically significant, χ^2^ = 86.9, p < .000 (Omnibus tests of model coefficient). The model explained 12.6% (Nagelkerke R^2^) of the variance in DSP behaviour and correctly classified 58.2% of cases. (Table 6) Collinearity statistics showed that there was no collinearity (tolerance and VIF of variables in the model ranged, 0.926–0.990 and 1.01–1.08).

**Table 6:** Predictors of DSP behaviour (binary logistic regression analysis).

Diagnosis	Wald	p
Aged below 30 years	4.6	0.033
Alcohol use disorder	10.4	0.001
Depressive disorder	18.8	0.000
Borderline Personality Disorder or traits	14.8	0.000

## Discussion

### Psychiatric morbidity and risk of DSH

The findings of this study show that one in seven patients with DSP was suffering from a major psychiatric disorder. One in twelve had a personality disorder or traits suggestive of personality disorder. However, the majority of patients (79.7%) presenting with DSP did not have any DSM-IV TR axis-I and/or II psychiatric diagnosis. The observed prevalence of psychiatric comorbidity of DSP was considerably lower than the previous findings in low and middle-income countries [17], Asian countries [10] and countries in the Indian subcontinent [12–15]. In general, Asian countries reported lower rates of psychiatric comorbidity compared to western countries, 70.6% vs 89.6% [10]. The use of a validated diagnostic interview may have contributed to observed lower rates as there is no overestimation possibly seen with the use of screening tools [17]. Most of the previous research in Asia has focused on the association of single psychiatric diagnosis [10] and are based on a retrospective diagnosis. A small proportion of studies was based on a standardized interview schedule-driven diagnosis [17]. Among those with psychiatric disorders, it is important to assess whether the distribution of psychiatric disorder and the associations with DSH are comparable to the non-Asian data and if so whether this has implications for treatment.

### Alcohol use disorders

Several studies have shown strong associations between alcohol and suicidal behaviour in Sri Lanka [21, 22]. The marked male predominance of alcohol use in this study, which has also been reported by the previous Sri Lankan studies and the rest of Asia [23], reflects the socio-cultural norms of alcohol use in this country. In this study, the presence of an alcohol use disorder was associated with 10 times increased risk of DSP. Alcohol use has been identified as the most significant risk factor associated with DSH for males [4]. This is supported by findings from other studies in Sri Lanka [24–26]. Interpersonal conflicts, domestic, physical, sexual or psychological abuses associated with alcohol misuse are likely to contribute to DSH behaviour [25]; both in the persons who misuse alcohol as well as in their close family members. Alcohol use and alcohol-related domestic violence among males are associated with an increased risk of psychiatric disorders among their partners and wives [27]. Alcohol use disorders are also associated with an increased risk of depression and suicidal behaviour among those who consume alcohol [28]. A recent study has proposed a model to explain suicide proneness in alcohol use through alcohol-related problems; negative life events and depressive symptoms, and multiple complex factors likely contribute to the increased risk of self-harm seen in those with alcohol use disorders in this study [29]. A meta-analysis reported a higher risk rate for alcohol use and suicide in western countries compared to Asian countries [30]. And, the reported pooled prevalence of alcohol use disorders in DSH is higher according to the systematic review, in which the majority of the included studies were conducted in the western countries [10]. The lower prevalence of alcohol use disorders among DSH in the presence of incremental consumption patterns [31] highlighted the importance of non-psychiatric precipitants.

Though the reported prevalence of other substance dependence is very low in the present study, a higher prevalence of cannabis has been reported in the previous studies [32, 33]; especially among adolescents [34]. Analysis of data published by Sri Lankan Police, from 2005 to 2015 ten years, showed that 2664 (6.3% of all suicide) suicides had concurrent psychoactive substance use [35]. The observed lower prevalence of other substance dependence may be due to the two main reasons; (1) prevalence of substance use higher compared to the substance use disorder, and (2) psychoactive substance use is more prevalent in urban areas compared to rural areas [36]. The involvement of psychoactive substances for suicide (12%) and DSH (8%) in low and middle-income countries are higher than the prevalence in Sri Lanka.

### Depression

In psychiatric disorders, mood disorders have been commonly [10, 17] and consistently [37] associated with suicidal behaviour. In this study too we found that having a depressive episode was associated with a 19 times higher risk for DSP. A few other studies conducted previously in Sri Lanka have reported depressive disorder as the most commonly seen psychiatric disorder among those who attempt DSH. Two previous local studies have reported a higher prevalence of depression among those who attempt DSH compared to the present prevalence of 8.4%, namely 27%—diagnosis obtained through a clinical assessment by a specialist [4], and, 51%—screened with PHQ-9 [38]. A recent meta-analysis reported a comparatively higher prevalence of mood disorders among DSH in low and middle-income countries, 21% [17]. A systematic review in which the majority of the included studies were conducted in western countries, reported a pooled prevalence of 49.4% for depressive disorder [10]. A higher prevalence rate was observed among females who were aged above 30 years than the males of the same age group, similar observations have been made in different settings [10]. The observation of a lower prevalence of depressive disorders in DSH in this study, compared to regional [39] and other low and middle-income countries, may be explained through the culture-specific contextual factors, such as DSH being a learned maladaptive coping response or a method of communicating distress [25]. Also of importance is our finding that less than a quarter of persons with depression who presented with DSH had been previously diagnosed–indicating the need for increased awareness and detection of depression in the community.

### Borderline personality disorder and borderline personality traits

A personality disorder is also a significant risk factor associated with suicidal behaviour [40]. Half to three-quarters of patients with borderline personality engage in suicidal behaviour and they have an average of three lifetime events of DSH [41, 42]. Previous studies conducted in Sri Lanka also cited that personality disorders, especially impulsive personality traits, were common among DSH patients [4, 43]. In this study in the KD, 8.7% of those who have presented with DSP were diagnosed as having borderline personality disorder or borderline personality traits, which is similar to the global estimated rates, 3–9% [44]. Previous work has been shown that specific recent life events (especially related to love, marriage, education), which are the main triggers for DSH in adolescents in rural Sri Lanka [25], may increase the imminent risk for suicidal behaviour among individuals with borderline personality disorder [45].

### Variation of psychiatric morbidity with age

It has been suggested that there may be significant differences between younger and older people who have suicidal behaviour [46]. Researchers have described two broad categories of persons who attempt DSH—a larger proportion of younger people, usually without psychiatric disorders, in response to family or social conflicts; and less commonly older persons, who often have an undiagnosed, major psychiatric disorder, especially depression, and who may have an ongoing high risk of suicide [4]. Similarly in this study too, older people (aged over 30 years) who presented with DSH were significantly more likely to have psychiatric morbidity, compared to younger persons. However, DSP was more common among younger people—being aged less than 30 years was an independent risk factor for DSP. A higher prevalence of adjustment disorder in younger people with DSH has been reported in several low-income settings [12, 47–49]–but this was not observed in this study. Alcohol use disorders and depressive disorders were the significant correlates of the self-harm behaviour in adults who were older than 30 years of age. The pattern of alcohol use disorder prevalence by age observed in the present study is similar to the pattern of alcohol use in Sri Lanka [31].

### Mental health based possible prevention strategies

Establishing a method of screening for psychiatric morbidity at least for all older persons (aged over 30 years) following an event of DSH, and further assessment as needed should be a part of routine practice. A significant finding of this study is that a large majority who presented with DSP with mental illnesses were diagnosed only after the attempt. This indicates an urgent need for increased awareness and detection of common psychiatric morbidity such as depression, in the community. Furthermore, healthcare practitioners, especially first contact healthcare workers, should be educated about the ‘red flags’ to identify high-risk persons presenting with DSP–for example, those with psychiatric morbidity such as depression or alcohol use disorder, those with previous attempts, or with significant ongoing life-stressors. Primary care doctors should receive training on the diagnosis and management of depression and alcohol use disorders.

### Non-psychiatric precipitants and future directions

The relatively low prevalence of psychiatric disorders seen in our prospective study is comparable to the prevalence of 32.1% found in a previous cross-sectional study conducted in the same district [4]. Other Sri Lankan studies conducted in socio-economically similar areas but utilizing different methodologies have also reported a low prevalence of psychiatric disorders [24, 43, 50, 51]. Regionally, the role of mental illness in suicide in Asia has been noted to be not as important as that seen in Western countries where the rates of Axis I disorder are as high as 98% of cases [52–55]. Other important differences between Asia and the West in DSH identified include the significantly lower repetition rates of self-harm, observed in Sri Lanka and Asia [7, 16].

These differences have very significant implications for public health planning including primary and secondary suicide prevention strategies. The lower prevalence of psychiatric diagnosis in Asia has been suggested to be possibly related to the differences in methodology and diagnostic tools used to elicit psychiatric disorders [52]. However, this explanation is not supported by the findings of our present prospective study, which uses a psychiatrist-administered DSM-IV-based SCID interview [56]. The importance of cultural factors is supported by the lower rates (23%) of psychiatric diagnoses seen in the minority ethnic groups in the UK [57].

In Sri Lanka, a greater focus on interventions that address non-psychiatric precipitants may be appropriate to reduce DSH. In rural Sri Lanka, DSP may be a method of dealing with difficult situations, particularly in the context of other background stressors, such as socio-economic difficulties [58]. Inability to cope with negative feelings, emotions and urges, and poor decision-making skills [59] and lack of necessary skills to deal with problems [4] have been identified as important in deliberate self-harm. Training on life skills including coping skills as a preventive strategy for suicide and DSH may be a worthy area to explore in future research [60–63]. Though the sensory processing patterns correlate with coping strategies, the relevance of these has not been established in Souths Asia [64–66].

## Limitations

The study was limited to DSP because this is overwhelmingly the most common method by which individuals present to the hospital with self-harm. A limitation is that we have not included those who self poisoned in the community, but who did not present to the hospital (likely to be low-lethality methods, such as self-cutting). Also, DSP is the predominant (> 80%) reason for DSH admission to the hospital [4], and the only cause to have sufficient numbers for study as part of a sub-study of educational interventions to improve the medical management of DSP. Though the current version of DSM is DSM-5, which has been published in May 2013 [67], the psychiatric assessment was conducted through SCID-IV and the diagnoses were presented according to the DSM-IV as it was the most updated version available during the study period. As medical comorbidity is rare among those who attempt DSP in rural Sri Lanka, a community-based control selection should have been done. However, due to challenges with feasibility, controls were selected from the ambulatory care hospital outpatients. The data was collected 7–9 years ago. A marked change in socioeconomic stress was not reported for the 2014 to 2019 period [68]. Restriction to access highly toxic pesticides was the most important public health intervention that has been implemented in Sri Lanka, over the past decade. Though these bans were associated with a reduction in suicide mortality [69], a persistently high incidence of DSP has been observed [5–7]. Hence, the underlying drives of DSP have probably remained the same and the present findings are applicable in practice. However, the possibility of changing the pattern of DSP due to the socio-economic changes and public health interventions can not be excluded.

## Conclusions

Depressive disorder and alcohol use disorders are significant predictors of DSP, especially for individuals aged above 30 years. The marked association between alcohol use disorders and DSP among males in this study highlights the urgent need for culturally suited effective strategies to minimize alcohol use in the community, including brief interventions and identification of high-risk individuals with alcohol use disorders requiring treatment. A majority of those with psychiatric disorders were diagnosed after the event of DSP. Hence, it is a missed opportunity for first-contact health care. While there was significant increased risk of DSP in the presence of a psychiatric disorder, the actual prevalence of psychiatric disorders among persons attempting DSP in rural Sri Lanka in this study was markedly low compared to the rates reported in the western and most of the non-western countries. Culture-specific non-psychiatric factors should be explored to explain the higher incidence rates and triggers for impulsivity. Further research is warranted to investigate whether DSH is the same phenomenon across the life cycle or whether it has a different meaning and risk factors in adolescence and young adults compared to the older individuals.
